# Antibacterial Activity of Green-Synthesized Silver Nanoparticles Using *Areca catechu* Extract against Antibiotic-Resistant Bacteria

**DOI:** 10.3390/nano11010205

**Published:** 2021-01-14

**Authors:** Jeong Su Choi, Hyon Chel Jung, Yeon Jae Baek, Bo Yong Kim, Min Woo Lee, Hyeong Dong Kim, Suhng Wook Kim

**Affiliations:** 1 School of Health and Environmental Science, College of Health Science, Korea University, Seoul 02841, Korea; guesschjs@korea.ac.kr (J.S.C.); wmitjhc@korea.ac.kr (H.C.J.); baekyeon@korea.ac.kr (Y.J.B.); erythro74@korea.ac.kr (B.Y.K.); leemw@korea.ac.kr (M.W.L.); hdkimx0286@korea.ac.kr (H.D.K.); 2 BK21 FOUR R&E Center for Learning Health Systems, Korea University, Seoul 02841, Korea

**Keywords:** green synthesis, silver nanoparticle, *Areca catechu*, antibiotic-resistant bacteria, antibacterial activity

## Abstract

In this work, the antibacterial activity of silver nanoparticles (AgNPs) synthesized using *Areca catechu* extracts against three species of antibiotic-susceptible and three species of resistant bacteria was investigated. The effects of this plant were more promising when compared with other medicinal plants tested. The hydrothermal extract of *Areca catechu* was mixed with silver nitrate to synthesize AgNPs. The synthesized particle characteristics were analyzed by UV–Vis spectrophotometry, scanning electron microscopy (SEM), dynamic light scattering (DLS), and Fourier-transform infrared spectroscopy (FT-IR). Minimum inhibitory concentration and minimum bactericidal concentration tests were conducted to confirm antibacterial activity and the results showed that AgNPs synthesized using *Areca catechu* extracts effectively inhibited the growth of bacterial species. Moreover, the SEM images of the bacterial species treated with AgNPs synthesized with *Areca catechu* extracts showed that clusters of AgNPs were attached to the surface of the bacterial cell wall, which could induce destruction of the cell membranes. The results suggest that AgNPs synthesized with *Areca catechu* extracts have the potential to treat antibiotic-resistant bacteria known as the major cause of nosocomial infections.

## 1. Introduction

Antibiotic-resistant bacteria are a worldwide problem that has emerged due to the misuse and long-term administration of antibiotics. Bacteria exposed to antibiotics can acquire resistance to antibiotics and transmit the resistance to other microorganisms, giving them the same properties. In addition, the resistance to one antibiotic may manifest as resistance to many other antibiotics that share the same structure, resulting in multidrug-resistant (MDR) strains. Recently, nosocomial infections caused by MDR bacteria have continued to increase and have emerged as an important health problem [1].

Nanotechnology can be applied as a new alternative to treating MDR bacteria. Nanotechnology is one of the most actively studied fields in medicine, biotechnology, and material engineering. Nanoparticles are small substances of 1 to 100 nm in size. They can provide solutions for technical problems such as next-generation energy use, environmental pollution, and medication for incurable diseases due to their physical and biological properties [2]. Specifically, silver is well known for its antibacterial activity, and silver nanoparticles (AgNPs) exert powerful antibacterial potential against both Gram-positive and Gram-negative bacteria [3].

Various methods have been devised to synthesize AgNPs by chemical, electrochemical, radiation, and photochemical techniques. However, AgNPs synthesized by physical and chemical methods can lead to environmental pollution, toxic effects on the human body, technological problems, high cost, and low efficiency [4,5]. To solve these problems, biological approaches that use ecofriendly and non-toxic chemicals in the synthetic process are gaining attention. The method that uses extracts from bioorganisms as reducing and capping agents to produce AgNPs is called green synthesis [6].

In this study, the hot water extract of various medicinal plants was used to synthesize AgNPs. In particular, the *Areca catechu* extract demonstrated good performance, so the characteristics of the synthesized particles were estimated by various experimental methods and antibacterial activity against antibiotic-resistant bacteria was confirmed.

## 2. Materials and Methods

### 2.1. Medicinal Plants

The medicinal plants in this study were purchased from a local medicinal herb market. They were covered tightly and stored in a cool and dry place to prevent oxidization. The plants used are shown in Table 1.

### 2.2. AgNP Synthesis Using Various Medicinal Plants

The medicinal plants were extracted by hydrothermal extraction. Distilled water (150 mL) with 15 g of the medicinal plant was boiled at 90 °C for five hours. The extract was centrifuged at 7000 rpm for 20 min and the supernatant was collected and filtered twice through Whatman No.1 filter paper. Next, the extract was filtered with a 0.2 µm syringe filter and stored in a deep freezer until used. The synthesis conditions were kept equal for all medicinal plant extracts. The concentration of the AgNO_3_ solution used for synthesis was 2 mM and the plant extract and AgNO_3_ were mixed at a ratio of 1:10. The mixture was incubated at room temperature for four hours in the dark. After that, the absorbance of all mixtures was measured at a range of 300–800 nm by a UV-Vis spectrophotometer (Thermo Fisher Scientific, Waltham, MA, USA).

### 2.3. Optimization of AgNP Synthesis Conditions Using A. catechu Extract

To determine the optimal conditions of AgNPs synthesis using *A. catechu* extract, three factors of AgNO_3_ concentration, mixing ratio, and reaction time were considered. These conditions are known to influence the synthesis of AgNPs using natural materials [7,8]. First of all, a fixed mixture ratio (volume of *A. catechu* extract:volume of AgNO_3_ solution, 1:50) was used to determine the adequate AgNO_3_ solution concentration for the synthesis and each mixture was incubated at room temperature for 4 h in the dark to prevent the photoactivation of AgNO_3_. The AgNO_3_ solution concentrations varied in 1 mM units from 1 to 10 mM. Then, a second experiment was conducted to determine the optimal mixture ratio of *A. catechu* extract using AgNO_3_ solution adjusted to the concentration determined in the test above. The mixture ratios were tested from 1:5 to 1:100 and each mixture was incubated at room temperature for 4 h in the dark. Finally, the synthesis of AgNPs was checked at incubation times of 15 min, 30 min, 1 h, 2 h, 3 h, and 4 h to confirm the time-dependent synthesis pattern under the conditions set by the two previous experiments. All results were analyzed by a UV-Vis spectrophotometer (Thermo Fisher Scientific, Waltham, MA, USA) in an absorbance range from 300 to 800 nm.

### 2.4. Characterization of the Synthesized AgNPs

The size and shape of the synthesized AgNPs were analyzed by scanning electron microscopy (SEM). The AgNP solution using *A. catechu* extract was spread on a cover glass and dehydrated in a dry oven for 24 h at 60 °C. Then, the dried cover glass was placed on a copper stub and attached with carbon tape. SEM images were acquired using a JEOL, Ltd. (Tokyo, Japan) instrument (JSM-6701F) at 10 KV accelerating voltage. The colloidal stability was evaluated by dynamic light scattering (DLS, Zetasizer Nano S90 System, Malvern, UK) by measuring the particle size distribution for 72 h.

A Fourier transform infrared (FT-IR) spectrophotometer (Perkin Elmer, Spectrum 100) was used to identify the presence of major functional groups of *A. catechu* extract affecting the synthesis of the AgNPs. The results were recorded over a range from 380 to 4000 m^−1^.

### 2.5. Well Diffusion Test of the AgNPs

The antibacterial activity of the AgNPs using *A. catechu* extract was screened by the well diffusion test. The bacterial strains of six species consisting of three antibiotic-susceptible species and three antibiotic-resistant species were tested. These antibiotic-susceptible bacteria were purchased from the Korean Collection for Type Cultures (KCTC) and antibiotic-resistant bacteria were purchased from the Culture Collection of Antimicrobial Resistant Microbes (CCARM). All the bacterial strains are shown in Table 2. Suspensions of the bacterial species with an optical density (OD) of 0.5 McFarland (1~2 × 10^8^ CFU/mL) units were uniformly swabbed on Mueller–Hinton agar (BD Difco, USA). Wells 6 mm in diameter were made with a sterile cork-borer and filled with various concentrations of AgNPs (360, 180, 90, 45, 22.5, and 11.25 μg/mL). The plates were incubated at 37 °C for 24 h and the diameter of the inhibition zone (mm) was measured to evaluate the antibacterial activity of the AgNPs. Vancomycin was used as a positive control against Gram-positive bacteria depending upon their antibiotic-resistant properties and gentamycin was used as a positive control against Gram-negative bacteria. Distilled water without AgNPs was used as a negative control. All experiments were performed three times and the results are shown as the mean (M) and standard deviation (SD).

### 2.6. Minimum Inhibitory Concentration and Minimum Bactericidal Concentration of the AgNPs

The analysis of minimum inhibitory concentration (MIC) and minimum bactericidal concentration (MBC) was performed in 96-well plates to determine the antibacterial activity of AgNPs against six bacterial strains. The MIC experiments were conducted according to the National Committee for Clinical Laboratory Standards M07-A09 (Clinical Laboratory Standards Institute, CLSI, 2012) broth microdilution method [9]. Mueller–Hinton broth (188 μL), 10 μL of bacterial suspension adjusted to 0.5 McFarland units, and 2 μL of AgNP solution were contained in each well and incubated at 37 °C for 24 h. The final concentration of AgNPs was serially diluted, ranging from 360 to 0.7 μg/mL. After incubation, the optical density at 620 nm was measured using a microplate reader (SpectraMax 190, Molecular Devices, USA). MIC_50_ was defined as the lowest concentration of AgNPs less than 50% optical density of the negative control.

For the MBC tests, each 50 μL mixture from the 96-well plates showing no visible growth in the MIC tests was sub-cultured on Mueller–Hinton agar at 37 °C for 24 h [10]. The lowest concentration with no growth on the agar was defined as the MBC. All of the experiments were conducted in triplicate.

### 2.7. Morphology of the Antibiotic-Resistant Bacteria Treated with AgNPs

The morphologic features of the antibiotic-resistant strains treated with AgNPs were observed by SEM. In the control wells, the suspensions of the bacterial species were adjusted to 0.5 McFarland units in Mueller–Hinton broth and inoculated into 6-well plates containing custom-made 20 × 20 mm polystyrene coverslips and incubated at 37 °C for 24 h. In contrast, the test wells were treated with AgNPs at the MIC. After incubation, the bacteria cultured on polystyrene coverslips were washed with phosphate-buffered saline (PBS) and fixed overnight in 2.5% glutaraldehyde in PBS. Then, the coverslips were washed with PBS to conduct the dehydration processes. Dehydration was accomplished in a graded series of ethanol (30%, 50%, 70%, 90%, 95%, and 100%) and the coverslips were dried for 24 h in a desiccator after dehydration. The dried samples were coated with platinum using an automatic magnetron sputter coating system (JEOL, Ltd., Tokyo, Japan) and the morphology of the bacteria was observed by SEM. The images in this paper are representative of the entire surface of the sample to show the effect of the AgNPs.

### 2.8. Statistical Analysis

For the antimicrobial activity of the AgNPs, all experiments were performed three times and the results are represented as the mean and standard deviation. All values were analyzed with Statistical Package for Social Scientists (SPSS) software (version 20., IBM Corp., Armonk, NY, USA).

## 3. Results and Discussion

### 3.1. AgNP Synthesis Using Various Medicinal Plants

**Figure 1:**
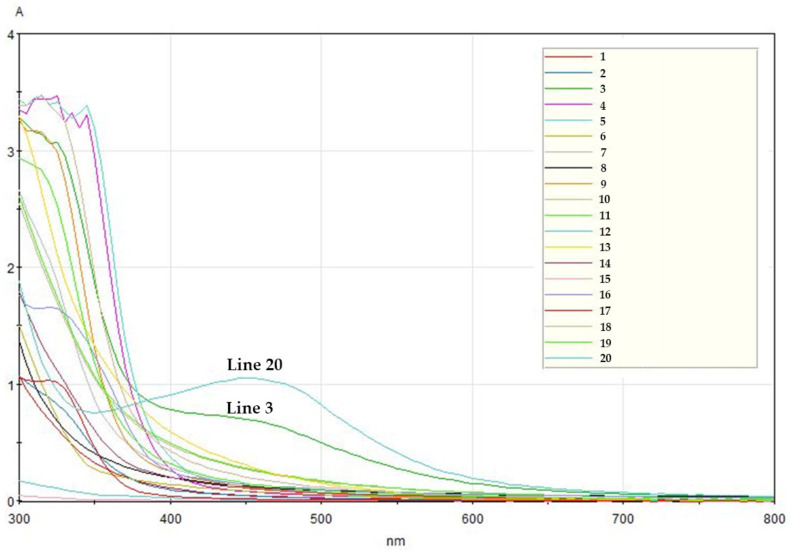
UV-Vis spectra of silver nanoparticles (AgNPs) synthesized with various medicinal plants. The line numbers in this figure follow the numbers in Table 1.

AgNP formation was confirmed using a UV-Vis spectrophotometer. According to other studies, noble metals such as gold and silver have unique optical properties because of their surface plasmon resonance (SPR), which is the collective oscillation phenomena of the conduction electrons [11]. It is well established that synthesized AgNPs exhibit a brown color due to surface plasmon vibration excitation, which shows a maximum absorbance peak between 430 and 450 nm. In Figure 1, *Areca catechu* extract (line 20) showed the most typical AgNP spectrum with a peak near 450 nm, suggesting that *Areca catechu* extract was the best for synthesizing AgNPs. *Xanthium sibiricum* extract (line 3) also showed an increase in absorbance at wavelengths around 450 nm but did not display the characteristic spectral pattern of AgNPs as much as *Areca catechu* extract. Moreover, other extracts were considered unsuitable for AgNP synthesis, given that they did not show characteristic spectral patterns.

### 3.2. Optimization of AgNP Synthesis Conditions Using Areca catechu Extract

**Figure 2:**
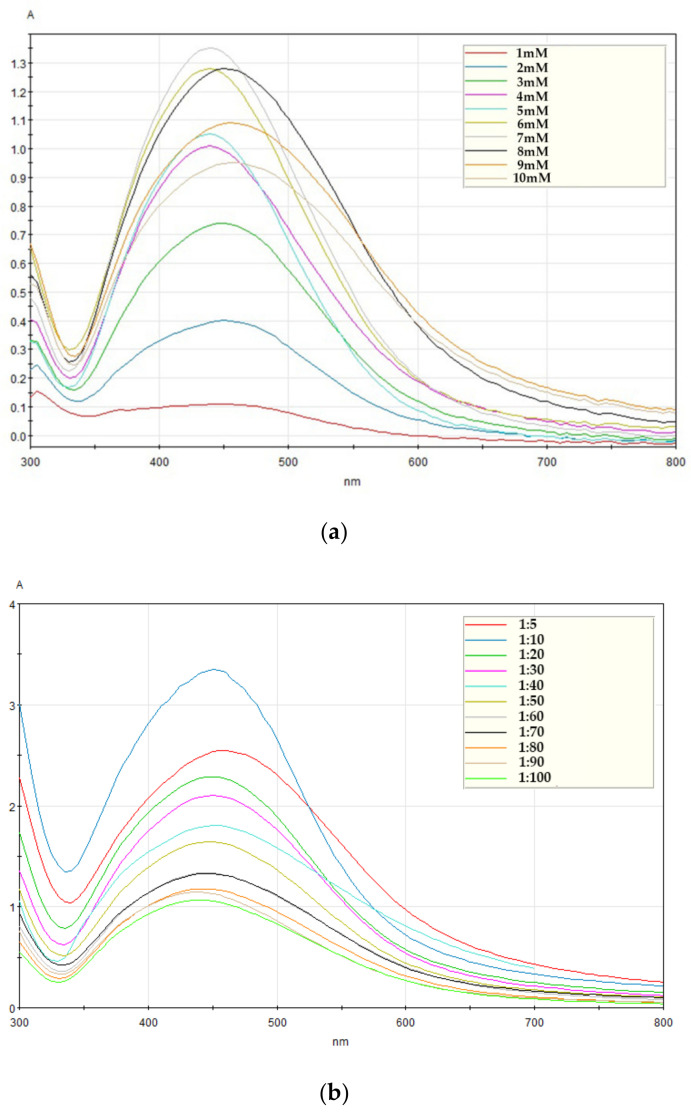

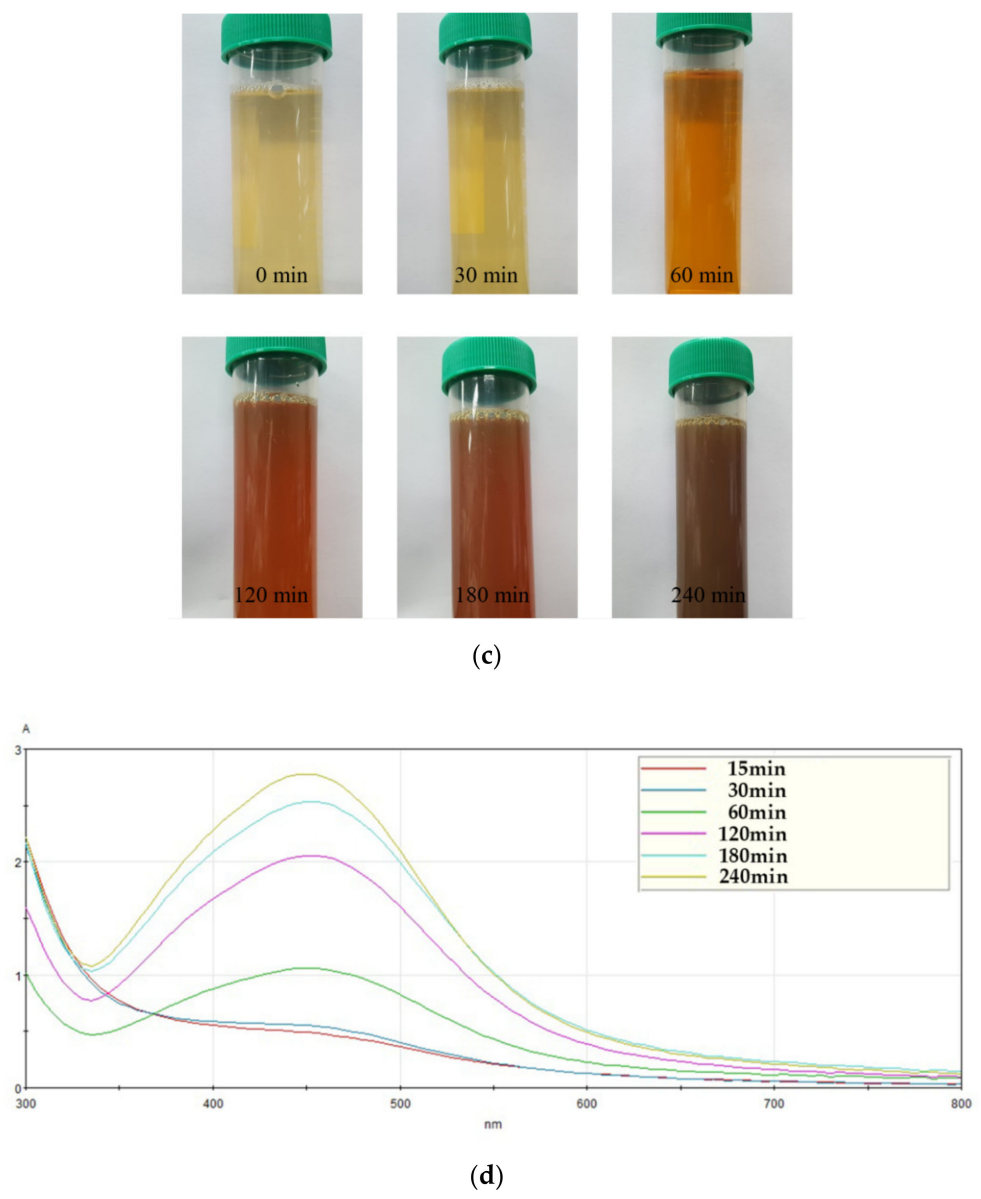
Absorbance spectra of AgNPs using *A. catechu* extract. (**a**) Absorbance spectra according to various AgNO_3_ solution concentrations, (**b**) absorbance spectra according to various mixing ratios, and (**c**) color changes in the mixture according to reaction time and (**d**) time-dependent absorbance patterns of the mixture (*A. catechu* extract:7 mM AgNO_3_ solution, 1:10).

With a fixed mixing ratio of 1:50 (*A. catechu* extract: AgNO_3_ solution), the absorbance was analyzed to find the concentration of AgNO_3_ solution that effectively formed AgNPs. Figure 2a shows that the spectral absorbance patterns gradually increased as the concentration increased, showing the highest absorbance of 7 mM AgNO_3_ solution at 430 nm. However, the spectral absorbance patterns were wider and lower at concentrations above 7 mM and the peak absorbance wavelength shifted toward 450 nm. The broadened peaks and shift toward longer wavelengths, known as red-shifting, indicated an increase in the particle size of the AgNPs in the colloidal solution [12]. These results indicated that the effective concentration for the synthesis of AgNPs was 7 mM.

The reaction mixture was analyzed with various mixing ratios from 1:5 to 1:100 (*A. catechu* extract:7 mM AgNO_3_) for the formation of AgNPs and the results are shown in Figure 2b. Since the highest peak absorbance was seen in the 1:10 ratio spectrum, the optimal mixing ratio was estimated at a 1:10 ratio. Finally, an experiment was conducted to determine the appropriate time of AgNP synthesis using 7 mM AgNO_3_ solution and a 1:10 mixing ratio obtained in the previous experiments. The color change of the mixture over time is revealed in Figure 2c, and this color change signified that the reduction of Ag^+^ ions occurred. The mixture of *A. catechu* extract and AgNO_3_ gradually changed in color from light yellow to reddish-yellow within one hour. After four hours of reaction time, the color was dark brown, indicating the synthesis of AgNPs. The time-dependent SPR pattern showing one specific peak was also observed by UV–Vis spectrophotometry (Figure 2d). The SPR pattern is established by the physical properties of the synthesized nanoparticles and interaction properties between the nanoparticles [13]. In this experiment, the peak wavelength was observed at 450 nm. The absorbance increased gradually over time, showing a maximum absorbance at four hours. The retention of the SPR patterns indicated the end of the synthesis reaction.

### 3.3. SEM Image and Particle Size Distribution of the Synthesized AgNPs

**Figure 3:**
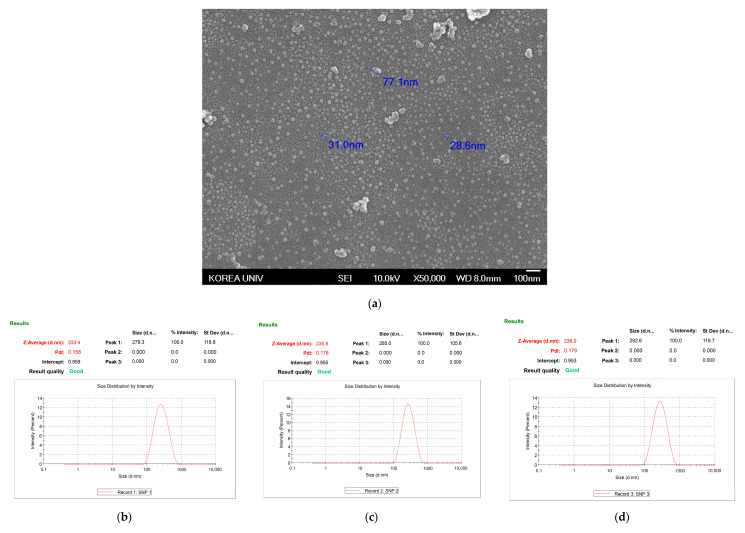
Particle properties of AgNPs using *A. catechu.* (**a**) Scanning electron microscopy (SEM) image and particle size distribution curves at (**b**) 24 h, (**c**) 48 h, and (**d**) 72 h.

SEM was used to confirm the shape and size of the formed AgNPs, and a representative image is shown in Figure 3a. According to other studies, the size and shape of AgNPs might vary depending upon the properties of the extracts used for synthesis. The average size of the AgNPs was 25 nm and the shapes were round when AgNPs were formed using *Boerhaavia diffusa* [14]. The particle size of AgNPs with leaves of *Datura metel* was 6–40 nm and the shapes showed quasilinear superstructures [15]. In this study, the particles were usually synthesized in the size range of about 20 to 30 nm, and a few 80 nm particles were synthesized by the aggregation of small particles. The shapes of the AgNPs were round, oval, and pebble-like, which appeared to be singular or agglomerate forms. This result suggested that the *A. catechu* extract could also play roles as reductant and capping agents to synthesize and scatter the AgNPs.

DLS was used to analyze the size distribution and colloidal stability of the AgNPs for 72 h (Figure 3). The z-average values were measured as 233.4 nm at 24 h (Figure 3b), 235.8 nm at 48 h (Figure 3c), and 238 nm at 72 h (Figure 3d). In addition, the polydispersity index (PDI) was measured at a low value of 0.179 after 72 h (Figure 3d), suggesting that the size distribution of the particles remained stable for 72 h.

### 3.4. FT-IR Analysis

**Figure 4:**
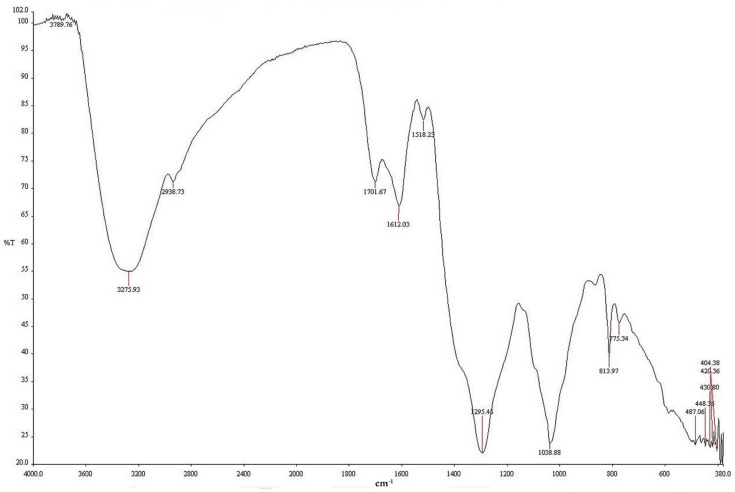
Fourier-transform infrared spectroscopy (FT−IR) spectrum of green-synthesized AgNPs using *A. catechu.*

FI-IR analysis was performed to identify the major chemical functional groups of *A. catechu* extract involved in the synthesis of the AgNPs (Figure 4). The FT-IR spectrum of green-synthesized AgNPs showed peaks at 3275, 2938, 1701, 1612, 1518, 1295, 1038, 813, and 775 cm^−1^. The strong peak at 3275 cm^−1^ may have resulted from the overlapping peaks of stretching vibrations of O-H and N-H groups [16]. Other peaks corresponded to C=H, C=O, aromatic nitro compounds, aromatic secondary amine, secondary alcohol, polysaccharides, and aliphatic chloro compounds. These are involved in natural compounds like alkaloids and polyphenols. *A. catechu* has been used as a herbal medicine for a long time, and includes many biomolecules such as alkaloids, tannins, flavones, triterpenes, steroids, and fatty acids [17]. These biomolecules might be considered to play key roles, affecting AgNP synthesis as reductant and capping agents.

### 3.5. Antimicrobial Activity of the Synthesized AgNPs

#### 3.5.1. Well Diffusion Assay

The antibacterial activity of the formed AgNPs was screened using the agar well diffusion assay. AgNPs serially diluted 2-fold from 360 μL/mL to 11.25 μL/mL were added to 6 mm wells on Muller–Hinton agar. The inhibition zones (mm) were measured to estimate the antibacterial effect of the AgNPs.

**Figure 5:**
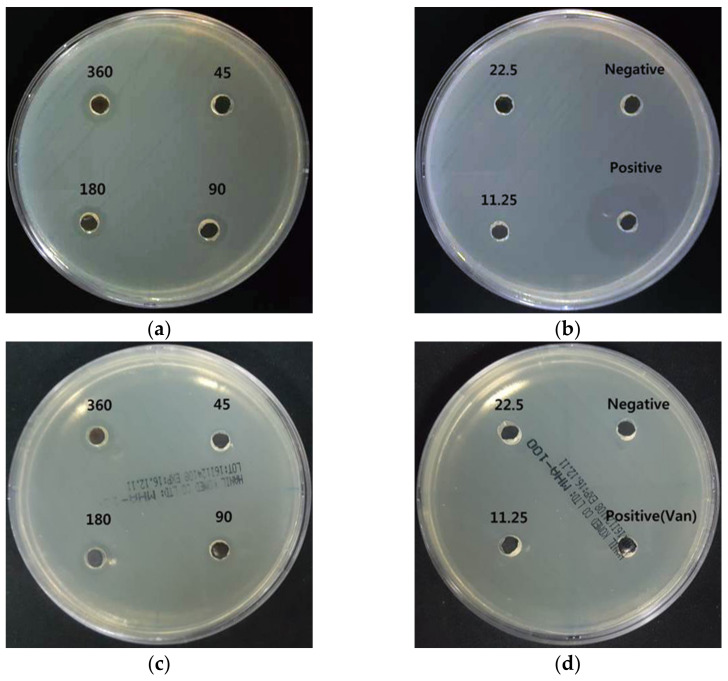
Antibacterial effects of AgNPs using *A. catechu* against *E. faecalis* and VRE. (**a**,**b**) Inhibition zone of AgNPs against *E. faecalis*; (**c**,**d**) inhibition zone of AgNPs against VRE. The AgNP treatment concentrations were 360, 180, 90, 45, 22.5, and 11.25 μg/mL.

*E. faecalis* belonging to *Enterococcus* spp. are Gram-positive cocci. *E. faecalis* is the most common nosocomial infection, often found in the bloodstream, urinary tract, and surgical sites, and is responsible for most enterococci infections in humans [18]. Vancomycin-resistant enterococci (VRE), which have spread worldwide, are recognized as a serious issue and various studies have been conducted on the organism [19]. In Figure 5, vancomycin used as a positive control had an effective antibacterial activity with a broad inhibition zone (27.0 ± 0.0 mm) for *E. faecalis*, but no inhibition zone was seen for VRE. AgNP treatment showed concentration-dependent inhibition zones in both bacteria. The AgNP inhibition zone data for *E. faecalis* and VRE are shown in Table 3.

**Figure 6:**
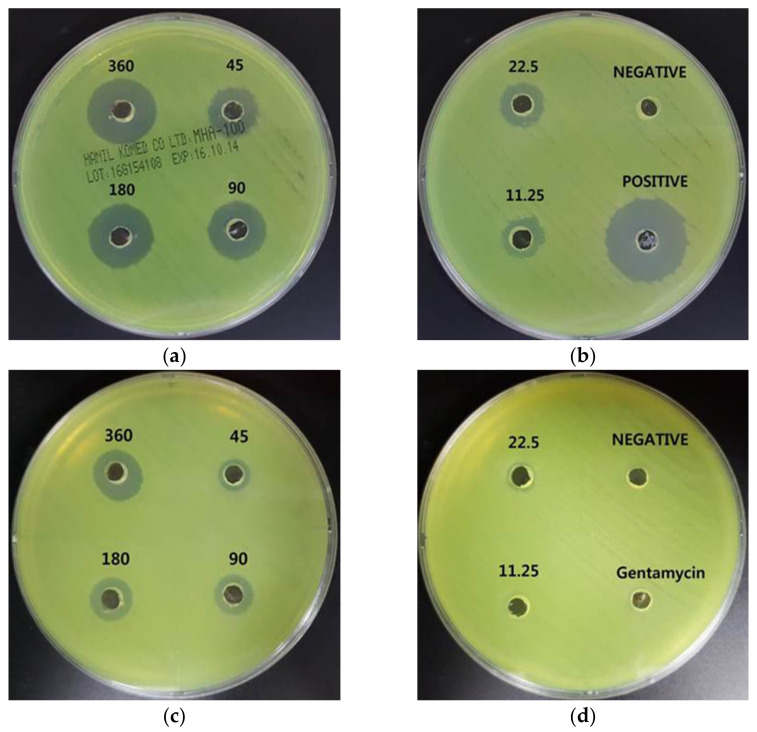
Antibacterial effects of AgNPs using *A. catechu* against *P. aeruginosa* and multidrug-resistant *Pseudomonas aeruginosa* (MRPA). (**a**,**b**): inhibition zone of AgNPs against *P. aeruginosa*. (**c**,**d**): inhibition zone of AgNPs against MRPA. The AgNP treatment concentrations were 360, 180, 90, 45, 22.5, and 11.25 μg/mL.

*P. aeruginosa* are Gram-negative bacilli, which exhibit green colonies due to a green pigment called pyocyanin. This species can cause chronic lung disease, meningitis, urinary tract infections, septicemia, and postoperative infection after organ transplantation [20,21]. The increase in multidrug-resistant *P. aeruginosa* (MRPA) is a critical public health problem that raises morbidity and mortality in hospitalized patients [22]. Gentamycin was used as a positive control for *P. aeruginosa* and MRPA, and Figure 6 shows that gentamycin formed a large inhibition zone (25.0 ± 0.7 mm) against *P. aeruginosa* but showed no inhibition zone in MRPA. AgNP treatment showed a slightly larger inhibition zone in *P. aeruginosa* than in MRPA but formed concentration-dependent inhibition zones for both bacteria. The results are shown in Table 4.

**Figure 7:**
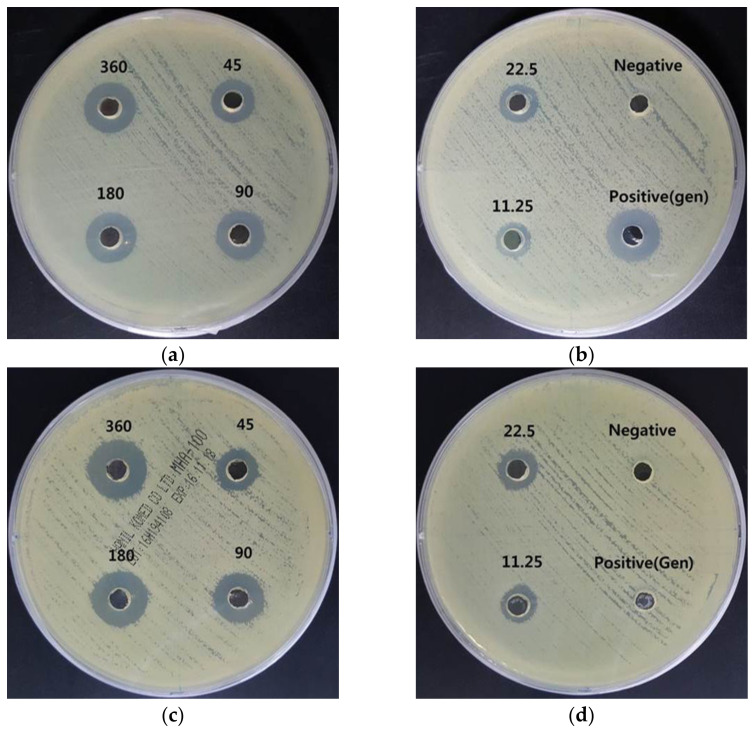
Antibacterial effects of AgNPs using *A. catechu* against *A. baumannii* and multidrug-resistant *Acinetobacter baumannii* (MRAB). (**a**,**b**): inhibition zone of AgNPs against *A. baumannii*. (**c**,**d**): inhibition zone of AgNPs against MRAB. The AgNP treatment concentrations were 360, 180, 90, 45, 22.5, and 11.25 μg/mL.

*A. baumannii* are Gram-negative coccobacilli with catalase-positive, oxidase-negative, non-mobility, and non-fermentation properties. In particular, it has been reported that *A. baumannii* can acquire resistance determinants to antibiotics. The outbreak of multidrug-resistant *A. baumannii* (MRAB) has increased the clinical significance of *A. baumannii* and indicated it as a threat to treatment with current antibiotics [23]. Figure 7 shows that *A. baumannii* had a 16.0 ± 0.0 mm inhibition zone formed by gentamycin as a positive control. However, MRAB did not show an inhibition zone from gentamycin. AgNP treatment formed concentration-dependent inhibition zones, representing effective antibacterial activity in both *A. baumannii* and MRAB. In particular, treatment with 360 μg/mL AgNPs resulted in a larger inhibition zone (*A. baumannii*: 16.8 ± 1.7 mm, MRAB: 17.7 ± 1.2 mm) than that of gentamycin (*A. baumannii*: 16.0 ± 0.0 mm, MRAB: 6.0 ± 0.0 mm). The inhibition zone data are shown in Table 5.

In addition, the antibacterial activity of the synthesized AgNPs was determined by MIC and MBC assays (Table 6). The broth dilution method was applied to the MIC assay for this study. MIC_50_ was defined as the minimum concentration inhibiting the growth of bacterial strains less than 50% compared to the absorbance of the negative control [24]. After 24 h of incubation, *E. faecalis*, VRE, exhibited an AgNP MIC value of 11.25 μg/mL. In contrast, the MIC of AgNPs for *P. aeruginosa*, MRPA, and *A. baumannii*, MRAB, was 5.6 μg/mL.

MBC was defined as the minimum concentration to completely inhibit the growth of strains on agar when they were sub-cultured after each MIC test. The results showed that the MBC value for most tested species except MRAB was 22.5 μg/mL, whereas MRAB had a lower MBC value at 11.25 μg/mL. The synthesized AgNPs were equally effective against both antibiotic-susceptible and resistant strains. Lara et al. reported that AgNPs had antibacterial properties regardless of antibiotic resistance and this feature was attributed to the bactericidal effect rather than bacteriostatic mechanisms [25].

#### 3.5.2. Observation of Antibiotic-Resistant Bacteria Treated with AgNPs Using SEM

**Figure 8:**
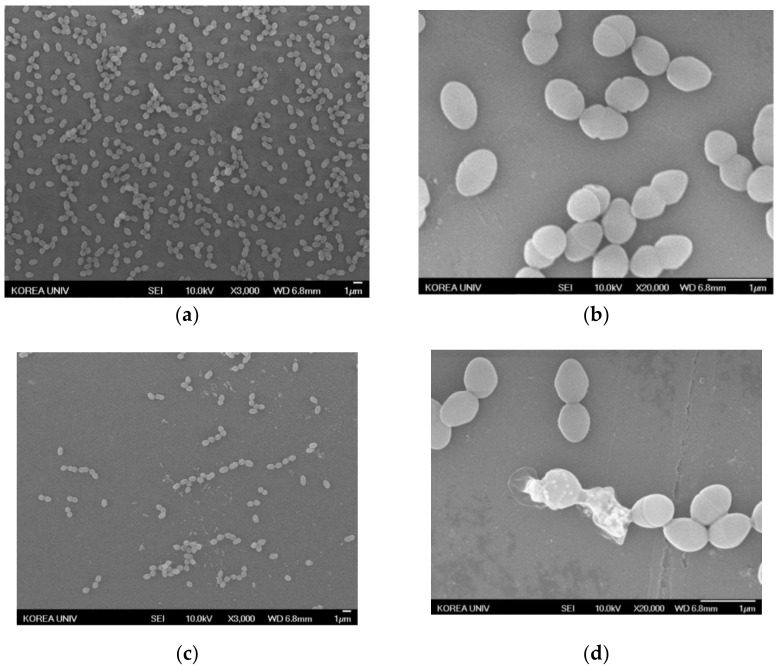
SEM of VRE treated with AgNPs. (**a**) Negative control magnified 3000×. (**b**) Negative control magnified 20,000×. (**c**) AgNP-treated VRE magnified 3000×. (**d**) AgNP-treated VRE magnified 20,000×.

**Figure 9:**
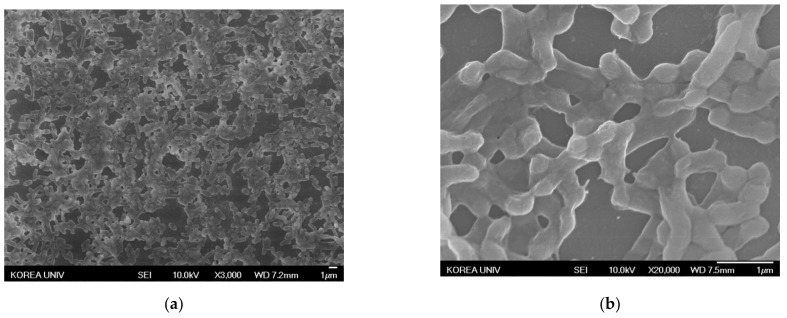

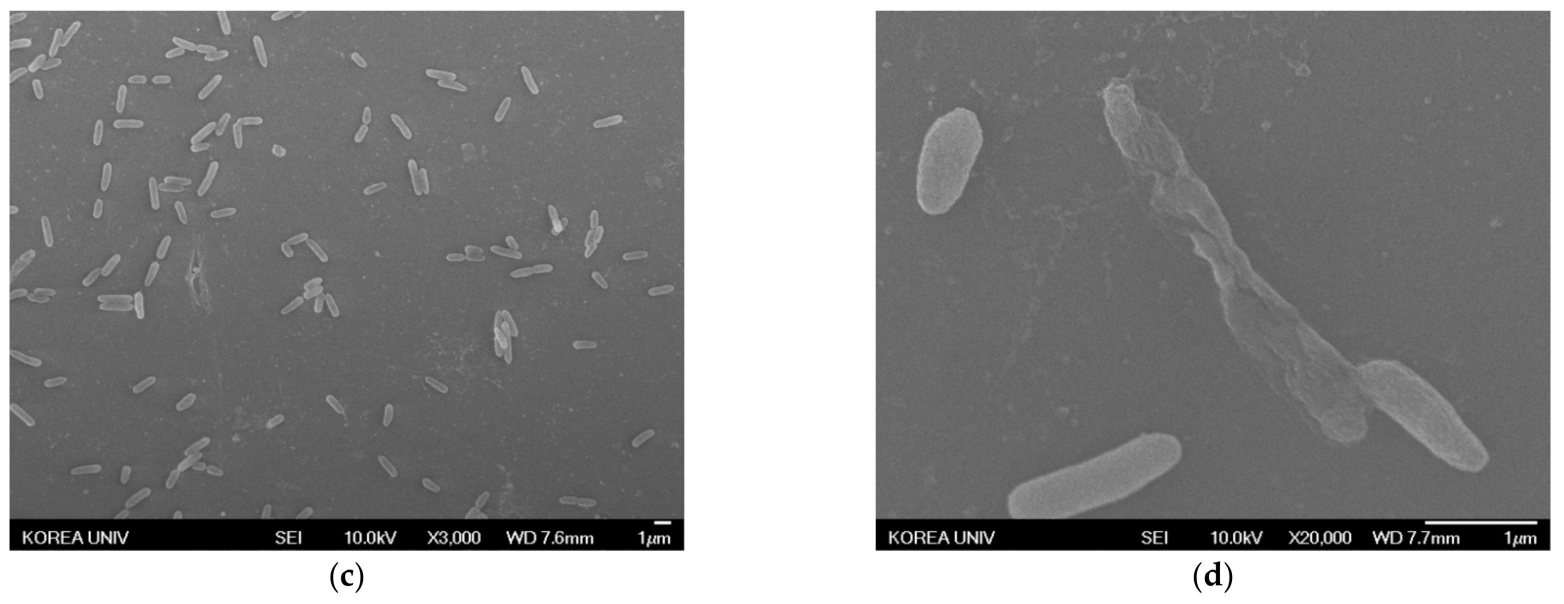
SEM images of MRPA treated with AgNPs. (**a**) Negative control magnified 3000×. (**b**) Negative control magnified 20,000×. (**c**) AgNP-treated MRPA magnified 3000×. (**d**) AgNP-treated MRPA magnified 20,000×.

**Figure 10:**
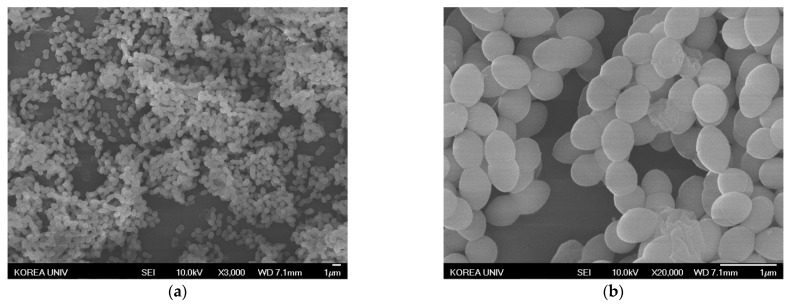

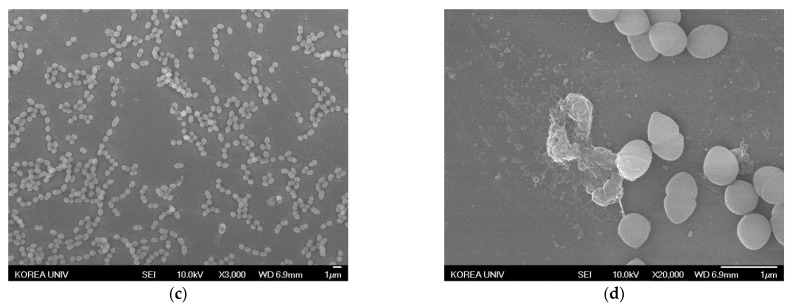
SEM images of MRAB treated with AgNPs. (**a**) Negative control magnified 3000×. (**b**) Negative control magnified 20,000×. (**c**) AgNP-treated MRAB magnified 3000×. (**d**) AgNP-treated MRAB magnified 20,000×.

Changes in morphology in antibiotic-resistant bacteria treated with AgNPs were observed by SEM. The resistant bacterial strains were treated with AgNPs at the MIC for 24 h. The images of the treated bacteria were compared to the controls not treated with AgNPs. The representative images are shown at magnifications of 3000× and 20,000× in Figure 8, Figure 9 and Figure 10. In contrast to the control (Figure 8a, Figure 9a and Figure 10a), the population of bacteria was obviously decreased in the 3000×-magnified images of all bacteria treated with AgNPs (Figure 8c, Figure 9c and Figure 10c). Especially, single AgNPs or clusters of AgNPs attached to the surface of the bacterial cell walls were observed in the 20,000×-magnified images of treated bacteria (Figure 8d, Figure 9d and Figure 10d) compared with control images (Figure 8b, Figure 9b and Figure 10b). The AgNP properties of direct adherence to bacterial cell membranes and subsequent bacterial destruction are the most well-known antibacterial mechanisms [26]. Another antibacterial mechanism of the AgNPs is that silver ions (Ag^+^) from the AgNPs disable the microbial growth processes. When AgNPs come into contact with moisture, the AgNPs elute Ag^+^ ions with antimicrobial activity [27]. These precedent studies suggested that AgNPs synthesized using *A. catechu extract* also exerted antibacterial activity through similar mechanisms.

## 4. Conclusions

The results of this study confirmed that *A. catechu* extract could be used to effectively synthesize AgNPs. This study also demonstrated that AgNPs produced with *A. catechu* extract had effective antibacterial activity demonstrated by the MIC test, MBC test, and SEM images. Thus, the potential use of AgNPs as a new antibiotic for treating antibiotic-resistant bacteria known as the major cause of nosocomial infections is suggested. AgNPs could also be an alternative to overcome the limitations of existing antibiotics. This is a more environmentally-friendly, human-friendly, and cost-effective method because *A. catechu*, a commonly used medicinal plant, can be used as both reductant and capping agents in the synthesis process without any other chemicals or harmful agents.

## Figures and Tables

**Table 1:** List of medicinal plants used for synthesis.

No.	Plants	Part Used
1.	*Curcuma zedoaria*	roots
2.	*Nelumbo nucifera Gaertner*	seeds
3.	*Xanthium sibiricum*	fruits
4.	*Polygala tenuifolia*	roots
5.	*Scutellaria baicalensis George*	roots
6.	*Cinnamomum cassia presl*	branches
7.	*Asiasarum sieboldii Miquel var*	leaves
8.	*Ophiopogon japonicus Ker-Gawler*	roots
9.	*Lindera glauca (Siebold & Zucc.) Blume*	branches
10.	*Sparganium stoloniferum Buchanan-Hamilton*	roots
11.	*Polygonatum sibiricum redoute*	roots
12.	*Poria cocos wolf*	roots
13.	*Atractylodes japonica Koidzumi*	roots
14.	*Torilis japonica*	seeds
15.	*Zizyphus jujuba Miller*	seeds
16.	*Dendropanax morbiferum Leveille*	roots
17.	*Aralia continentalis kitagawa*	leaves
18.	*Angelica dahurica*	roots
19.	*Paeonia japonica*	roots
20.	*Areca catechu*	fruits

**Table 2:** List of microorganisms used for antimicrobial experiments.

Bacteria	Strain
*Enterococcus faecalis* (*E. faecalis*)	KCTC 3206
Vancomycin-resistant *Enterococcus faecalis* (VRE)	CCARM 5025
*Pseudomonas aeruginosa* (*P. aeruginosa*)	KCTC 1637
Multidrug-resistant *Pseudomonas aeruginosa* (MRPA)	CCARM 2092
*Acinetobacter baumannii* (*A. baumannii*)	KCTC 2508
Multidrug-resistant *Acinetobacter baumannii* (MRAB)	CCARM 12005

**Table 3:** Diameters of inhibition zones of AgNPs using *A. catechu* against *E. faecalis* and vancomycin-resistant *Enterococcus faecalis* (VRE).

AgNP Concentration (μg/mL)	Inhibition Zone (mm)	
	*E. faecalis*	*VRE*
360	12.0 ± 0.9	12.3 ± 0.8
180	11.3 ± 0.9	11.2 ± 0.3
90	10.5 ± 0.5	10.1 ± 0.2
45	9.9 ± 0.3	9.2 ± 0.4
22.5	7.0 ± 0.3	6.0 ± 0.0
11.25	6.0 ± 0.0	6.0 ± 0.0
Positive control	27.0 ± 0.0	6.0 ± 0.0
Negative control (D.W.)	6.0 ± 0.0	6.0 ± 0.0

**Table 4:** Diameter of inhibition zones of AgNPs using *A. catechu* against *P. aeruginosa* and MRPA.

AgNP Concentration (μg/mL)	Inhibition Zone (mm)	
	*P. aeruginosa*	MRPA
360	19.8 ± 1.3	16.3 ± 1.5
180	18.4 ± 0.7	15.1 ± 0.8
90	17.5 ± 0.8	13.1 ± 0.3
45	15.2 ± 0.7	11.7 ± 0.6
22.5	12.8 ± 0.4	8.7 ± 0.3
11.25	10.7 ± 0.5	6.4 ± 0.5
Positive control	25.0 ± 0.7	6.0 ± 0.0
Negative control (D.W.)	6.0 ± 0.0	6.0 ± 0.0

**Table 5:** Diameter of inhibition zones of AgNPs using *A. catechu* against *A. baumannii* and MRAB.

AgNP Concentrationš (μg/mL)	Inhibition Zone (mm)	
	*A. baumannii*	MRAB
360	16.8 ± 1.7	17.7 ± 1.2
180	16.2 ± 1.3	16.9 ± 0.7
90	15.1 ± 0.5	15.7 ± 0.8
45	13.9 ± 0.5	13.4 ± 0.5
22.5	10.4 ± 0.5	12.2 ± 0.3
11.25	8.6 ± 0.7	10.5 ± 0.5
Positive control	16.0 ± 0.0	6.0 ± 0.0
Negative control (D.W.)	6.0 ± 0.0	6.0 ± 0.0

**Table 6:** Minimum inhibitory concentration (MIC) and minimum bactericidal concentration (MBC) results of AgNPs using *A. catechu* against 6 strains of bacteria.

Bacterial Strains	AgNPs	
	MIC (μg/mL)	MBC (μg/mL)
*E. faecalis*	11.25	22.5
VRE	11.25	22.5
*P. aeruginosa*	5.6	22.5
MRPA	5.6	22.5
*A. baumannii*	5.6	22.5
MRAB	5.6	11.25

## Data Availability

The data presented in this study are available on request from the corresponding author.
